# Effects of photobiomodulation combined with orofacial myofunctional therapy on the quality of life of individuals with temporomandibular disorder

**DOI:** 10.1590/2317-1782/20212020313

**Published:** 2022-04-08

**Authors:** Wellyda Cinthya Félix Gomes da Silva Dias, Renata Veiga Andersen Cavalcanti, Hipólito Virgílio Magalhães, Leandro de Araújo Pernambuco, Giorvan Ânderson dos Santos Alves

**Affiliations:** 1 Programa Associado de Pós-graduação em Fonoaudiologia, Universidade Federal da Paraíba – UFPB - João Pessoa (PB), Brasil.; 2 Programa Associado de Pós-graduação em Fonoaudiologia, Universidade Federal do Rio Grande do Norte - UFRN - Natal (RN), Brasil.

**Keywords:** Temporomandibular Joint Disorders, Quality of Life, Laser Therapy, Myofunctional Therapy, Speech, Language and Hearing Sciences

## Abstract

**Purpose:**

To analyze the impact of photobiomodulation combined with orofacial myofunctional therapy (OMT) on the oral health quality of life (OHQOL) of individuals with temporomandibular disorder, before and after the treatment.

**Methods:**

Blind, controlled, randomized clinical trial with 34 volunteers randomly distributed into two groups: G1, who received OMT combined with photobiomodulation, and G2, treated with OMT combined with inactive laser. The subjects were first assessed with a visual analog scale (VAS) to classify them according to the degree of orofacial pain and with the Oral Health Impact Profile – Short Form (OHIP-14) regarding the impact on the OHQOL. The resulting data were statistically analyzed. The significance level was set at 0.05 (95%).

**Results:**

“Physical pain”, “psychological discomfort”, “physical disability”, and “psychological disability” were the aspects with the greatest impact on the OHQOL. The G1 subjects responded positively to their treatment, as well as G2 to theirs. There was a strong positive correlation between VAS and total OHIP-14 score in both groups after the treatment. However, the functional recovery in the control group individuals (G2) was the most perceived positive change in the OHQOL in comparison with the experimental group (G1).

**Conclusion:**

The people who received photobiomodulation combined with OMT perceived an improvement in the OHQOL, as well as those treated with placebo laser. There was a strong positive correlation in both groups in the improvement of the degree of pain and self-perception of the OHQOL.

## INTRODUCTION

The temporomandibular disorder (TMD) is conceived by the American Academy of Orofacial Pain (AAOP) as a series of clinical conditions involving the masticatory muscles, the temporomandibular joint (TMJ), and the associated structures of the stomatognathic system^(1)^.

It manifests as various signs and symptoms. Pain is the main characteristic reported by subjects affected by this condition and it is sometimes the main reason why they seek treatment^(2)^. Hence, TMD is considered the main cause of nondental pain in the orofacial region^(3)^. The pain manifests mostly in the TMJ region, face, head, neck, and ears, and when opening and closing the mandible. It is associated with limited mandible movements, joint noises, and difficulties speaking, chewing, and swallowing^(4)^. Moreover, patients commonly report increased pain/discomfort when performing these functions^(5)^.

Thus, these problems have a direct and negative influence on the patients’ physical and mental health, affecting their school, professional, and social activities, even causing affective and cognitive imbalance^(6)^. Therefore, there are harmful consequences to these people’s quality of life (QOL) and particularly to their oral health quality of life (OHQOL) – with greater impairments depending on the severity of the TMD^(7)^.

This reinforces the awareness that TMD subjects need comprehensive clinical attention, as many aspects are involved. This is especially the case of the QOL, which is associated with the patients’ subjective perception of their position in life, in the context of the values and culture in which they live and in relation to their goals, expectations, and concerns. Hence, investigating the degree of impairment of the QOL is highly important to the health professionals who treat them^(4)^.

In many cases, though, the patients’ perceptions and feelings regarding oral health are ignored. Furthermore, few studies in the literature analyze the impact of the TMD painful conditions on the QOL, in contrast with the number of publications on TMD diagnosis, signs, and symptoms^(8)^.

Given this context, various therapeutic approaches have been proposed to ease and treat the consequences of the TMD clinical conditions and provide more comfort to the patients^(4)^. One of them is the orofacial myofunctional therapy (OMT), which highlights the work of the speech-language-hearing therapist in the field of oral-motor function. Its purpose is to provide orofacial myofunctional stability^(9)^ by changing the muscle and functional patterns^(10)^, thus easing their pain^(11)^, adjusting mandibular movement amplitude^(12)^, and consequently readjusting the speech, breathing, chewing, and swallowing functions^(10)^.

The use of low-level laser (LLL) or photobiomodulation in TMD has also been described and discussed in scientific research. It is characterized as a nonpharmaceutical, noninvasive, quick, and safe intervention, with favorable reactions in myogenic and joint pains, under the analgesic, anti-inflammatory, and biomodulator effects of the physiological cell functions^(13,14)^.

Authors point out that these effects positively ease the pain^(15)^, increase the mouth opening amplitude^(16)^, and aid in the signs, symptoms, and functional recovery^(17)^. On the other hand, some meta-analysis studies^(13,18)^ demonstrate that groups submitted to active laser had their pain eased likewise the placebo groups, although the first one had better results in the subjects’ functional status^(18)^.

Researchers^(17)^ further reinforce that combining treatments to control the pain and train the oral-motor functions suggests favorable TMD rehabilitation. Hence, the objective of this study was to analyze the impact on the OHQOL of subjects with TMD, before and after the photobiomodulation treatment combined with OMT, and verify possible correlations between the degree of orofacial pain and their self-perception of this impact.

## METHODS

This is a therapeutic intervention study designed as a longitudinal, blind, controlled, randomized clinical trial. It was approved by the Research Ethics Committee of the Center for the Health Sciences at the *Universidade Federal da Paraíba* (Federal University of Paraíba – UFPB), under report number 3.354.075, complying with the ethical principles of Resolution no. 466/2012, of the National Health Council (CNS, in Portuguese).

The research was conducted at the Speech-Language-Hearing Teaching Clinic of UFPB, involving participants of both sexes. The sample comprised 34 volunteers who sought the speech-language-hearing services on their own initiative.

Based on screenings, the eligibility criteria were as follows: subjects aged 18 to 59 years; with orofacial pain characterized as muscular TMD, according to the Research Diagnostic Criteria for Temporomandibular Disorders (RDC/TMD), Axis I^(19)^; with time available to undergo the treatment; and who agreed to participate in the study by signing the informed consent form. Those who reported a history of neurological impairment; malformations, tumors, traumas, or surgeries on the head and neck region; use of dentures or orthodontic appliances; who had already been submitted to or were at the time undergoing speech-language-hearing therapy; or who were taking myorelaxant and/or anti-inflammatory drugs or had taken them for more than one year were excluded.

Besides these factors, criteria that contraindicate photobiomodulation were also considered, namely: pregnant women; photosensitive patients; subjects with glaucoma; with a pacemaker or another electronic implant; and/or infected tissue near the irradiation site.

Before collecting the research data, the participants were randomly divided into groups in a simple draw. This random distribution formed G1 – Experimental Group, which received the OMT combined with photobiomodulation; and G2 –Positive Control Group, which received OMT combined with inactive laser (placebo).

The study participants were assessed before and after the period of therapeutic intervention, with the following procedures: Assessment of the impact on the OHQOL with the Oral Health Impact Profile – Short Form (OHIP-14) and of the degree of orofacial pain with a visual analog scale (VAS).

The OHQOL was analyzed with the OHIP-14 questionnaire, developed by Slade (1997), in its version already translated and validated in Brazilian Portuguese^(20)^. It was administered in an interview, considering the patients’ subjective perception of their problem over the last 6 months.

The instrument has 14 questions – two in each of its seven domains (functional limitation, physical pain, psychological discomfort, physical disability, psychological disability, social disability, and handicap). Each question has five answer options: never, rarely, sometimes, recurrently, and always. Following the sum method^(21)^, their scores are respectively 0, 1, 2, 3, and 4 points. The score in each domain ranges from 0 to 8 points, and higher scores mean greater impairment. As for the total OHIP-14 score, the sum of the ordinal answers ranges from 0 to 56 points – meaning a greater negative impact on oral health.

The VAS was administered with a non-numbered 10-cm line. The left end meant they reported “no pain” and the right end, “the worst pain possible” – i.e., the more the marking was made toward the left end, the better the classification of their degree of pain, whereas the more to the right, the worse this classification.

The volunteers were informed of how to fill in the scale, and then they made the self-analysis of spontaneous pain, marking the point on the line that best represented the degree of pain they felt at the moment of the assessment. To analyze the result, the examiner used a ruler under the line to identify, from 0 to 10, the number they indicated and transcribed the result to the patient’s clinical evolution form.

The intervention consisted of weekly meetings, totaling 13 sessions. One session was used to assess them and administer the said instruments; another one was used exclusively to instruct them about their clinical conditions, harmful habits that trigger the pains, and how to ease them at home; 10 sessions were used for the therapy itself; and yet another one was used specifically for reassessment. Each speech-language-hearing visit lasted 45 minutes – 30 minutes for the OMT and 15 and 12 minutes, respectively, in the first and second phase to apply the LLL. The team was made up of four calibrated researchers, trained in photobiomodulation and with 3-year experience in the necessary procedures.

The OMT speech therapy protocol^(9)^ was the same for both groups. It consisted of strategies such as thermotherapy and massage therapy, with slow and deep massages to favor local blood circulation and ease the pain. It also used myotherapy, with isometric and isotonic exercises for specific muscle groups (lips, tongue, cheeks, and mandibular muscles) to improve the flexibility, coordination, symmetry, and stability of the TMJ functional movements. Moreover, it trained their orofacial functions – i.e., using the masticatory function as exercise and training.

The active LLL corresponded to the bilateral application of aluminum/gallium arsenide (Al/GaAs) LLL with the Laser Pulse Line equipment, manufactured by IBRAMED. The protocol used was developed from discussions at the Center for Language Studies and Stomatognathic Functions (NELF, in Portuguese). It consisted of the application on five sites of the TMJ region (lateral pole and the superior, anterior, posterior, and inferior points of the condylar position) and on the painful points of the masseter, temporal, sternocleidomastoid, and trapezius muscles, as reported by the individuals.

The application used 830-nm wavelength (infrared) and irradiation with two different purposes in phases: the first phase (from the first to the fifth session), with doses of 6 J and fluence of 51 J/cm^2^, to ease the painful condition; and the second phase (beginning at the sixth session), with doses of 4 J and fluence of 34 J/cm^2^, to biostimulate the functional gains furnished by the speech-language-hearing therapy. The inactive LLL was simulated, providing the placebo effect.

The resulting data were tabulated and stored in a Microsoft Excel 2013 spreadsheet to be managed afterward. Both descriptive and inferential statistical analyses were made in the Statistical Package for the Social Sciences (SPSS) for Windows, version 21.0. In the descriptive analysis, approaching the qualitative variables, the absolute and relative (percentage) frequencies were applied; and, to address the quantitative variables, the mean and median were used as the measures of central tendency, and the standard deviation and quartiles 1 and 3 as the measures of variability.

As for the inferential analysis, the Wilcoxon test was used to analyze the intragroup relationship of the quantitative and qualitative variables, and the Mann-Whitney test for that between the groups. The Spearman correlation test was used to verify the correlation between the quantitative data. The results of this test are presented in percentages and may have positive and negative values. The degree of correlation was considered weak when the resulting value was up to 0.3; moderate, when between 0.3 and 0.7; and strong, when above 0.7^(22)^. The significance level in this study was set at 0.05 (95%).

## RESULTS

This study assessed and treated 34 individuals diagnosed with muscular TMD. In G1, there were 16 (84.2%) females and 3 (15.8%) males, with a mean age of 32.16±8.60 years. In G2, there were 11 (73.3%) females and 4 (26.7%) males, with a mean age of 34.67±13.05 years. The sample characterization, based on the subjective perception of the OHQOL before and after the treatment as indicated in each OHIP-14 question, is shown in Table 1.

**Table 1:** Absolute and relative distribution of the participants regarding the subjective perception of the oral health quality of life before and after the treatment. João Pessoa, Paraíba, Brazil. 2020

**OHIP-14 DOMAINS**	**QUESTIONS**	**ANSWERS**	**BEFORE THE TREATMENT**		**AFTER THE TREATMENT**	
			**G1**	**G2**	**G1**	**G2**
			**n (%)**	**n (%)**	**n (%)**	**n (%)**
**Functional limitation**	1. Have you had trouble pronouncing any words because of problems with your teeth, mouth, or dentures?	Never	10 (52.6)	10 (66.7)	12 (63.2)	15 (100.0)
		Rarely	1 (5.3)	1 (6.7)	1 (5.3)	0 (0.00)
		Sometimes	4 (21.1)	3 (20.0)	6 (31.6)	0 (0.0)
		Recurrently	2 (10.5)	1 (6.7)	0 (0.0)	0 (0.0)
		Always	2 (10.5)	0 (0.0)	0 (0.0)	0 (0.0)
	2. Have you felt that your sense of taste has worsened because of problems with your teeth, mouth, or dentures?	Never	14 (73.7)	9 (60.0)	15 (78.9)	15 (100.0)
		Rarely	1 (5.3)	2 (13.3)	3 (15.8)	0 (0.0)
		Sometimes	2 (10.5)	3 (20.0)	1 (5.3)	0 (0.0)
		Recurrently	0 (0.0)	1 (6.7)	0 (0.0)	0 (0.0)
		Always	2 (10.5)	0 (0.0)	0 (0.0)	0 (0.0)
**Physical pain**	3. Have you had painful aching in your mouth?	Never	2 (10.5)	0 (0.0)	7 (36.8)	7 (46.7)
	3.	Rarely	0 (0.0)	3 (20.0)	4 (21.1)	2 (13.3)
	3.	Sometimes	6 (31.6)	3 (20.0)	4 (21.1)	3 (20.0)
	3.	Recurrently	5 (26.3)	5 (33.3)	4 (21.1)	2 (13.3)
	3.	Always	6 (31.6)	4 (26.7)	0 (0.0)	1 (5.9)
	4. Have you found it uncomfortable to eat any foods because of problems with your teeth, mouth, or dentures?	Never	0 (0.0)	3 (20.0)	7 (36.8)	6 (40.0)
	3.	Rarely	3 (15.8)	1 (6.7)	3 (15.8)	3 (20.0)
	3.	Sometimes	1 (5.3)	1 (6.7)	5 (26.3)	3 (20.0)
	3.	Recurrently	6 (31.6)	3 (20.0)	3 (15.8)	1 (6.7)
	3.	Always	9 (47.4)	7 (46.7)	1 (5.3)	2 (13.3)
**Psychological discomfort**	5. Have you been self-conscious because of your teeth, mouth, or dentures?	Never	1 (5.3)	0 (0.00)	6 (31.6)	6 (40.0)
		Rarely	1 (5.3)	2 (13.3)	6 (31.6)	1 (6.7)
		Sometimes	5 (26.3)	4 (26.7)	3 (15.8)	6 (40.0)
		Recurrently	3 (15.8)	3 (20.0)	2 (10.5)	2 (13.3)
		Always	9 (47.4)	6 (40.0)	2 (10.5)	0 (0.0)
	6. Have you felt tense because of problems with your teeth, mouth, or dentures?	Never	0 (0.0)	3 (20.0)	5 (26.3)	5 (33.3)
		Rarely	0 (0.0)	1 (6.7)	4 (21.1)	3 (20.0)
		Sometimes	6 (31.6)	2 (13.3)	4 (21.1)	3 (20.0)
		Recurrently	4 (21.1)	5 (33.3)	3 (15.8)	2 (13.3)
		Always	9 (47.4)	4 (26.7)	3 (15.8)	2 (13.3)
**Physical disability**	7. Has your diet been unsatisfactory because of problems with your teeth, mouth, or dentures?	Never	2 (10.5)	2 (13.3)	11 (57.9)	8 (53.3)
		Rarely	0 (0.0)	0 (0.0)	2 (10.5)	0 (0.0)
		Sometimes	5 (26.3)	6 (40.0)	4 (21.1)	7 (46.7)
		Recurrently	5 (26.3)	4 (26.7)	2 (10.5)	0 (0.0)
		Always	7 (36.8)	3 (20.0)	0 (0.0)	0 (0.0)
	8. Have you had to interrupt meals because of problems with your teeth, mouth, or dentures?	Never	3 (15.8)	5 (33.3)	10 (52.6)	10 (66.7)
		Rarely	3 (15.8)	4 (26.7)	7 (36.8)	2 (13.3)
		Sometimes	6 (31.6)	2 (13.3)	2 (10.5)	3 (20.00)
		Recurrently	4 (21.1)	3 (20.0)	0 (0.0)	0 (0.0)
		Always	3 (15.8)	1 (6.7)	0 (0.0)	0 (0.0)
**Psychological disability**	9. Have you found it difficult to relax because of problems with your teeth, mouth, or dentures?	Never	1 (5.3)	2 (13.3)	5 (26.3)	5 (33.3)
	1.	Rarely	1 (5.3)	3 (20.0)	5 (26.3)	2 (13.3)
	1.	Sometimes	4 (21.1)	6 (40.0)	5 (26.3)	4 (26.7)
	1.	Recurrently	6 (31.6)	1 (6.7)	4 (21.1)	1 (6.7)
	1.	Always	7 (36.8)	3 (20.0)	0 (0.0)	3 (20.0)
	10. Have you been a bit embarrassed because of problems with your teeth, mouth, or dentures?	Never	10 (52.6)	8 (53.3)	12 (63.2)	13 (86.7)
		Rarely	1 (5.3)	1 (6.7)	6 (23.6)	0 (0.0)
		Sometimes	5 (26.3)	3 (20.0)	1 (5.3)	2 (13.3)
		Recurrently	0 (0.0)	2 (13.3)	0 (0.0)	0 (0.0)
		Always	3 (15.8)	1 (6.7)	0 (0.0)	0 (0.0)
**Social disability**	11. Have you been a bit irritable with other people because of problems with your teeth, mouth, or dentures?	Never	9 (47.4)	7 (46.7)	8 (42.1)	10 (66.7)
		Rarely	2 (10.5)	2 (13.3)	3 (15.8)	1 (6.7)
		Sometimes	2 (10.5)	0 (0.0)	4 (21.1)	1 (6.7)
		Recurrently	1 (5.3)	4 (26.7)	4 (21.1)	3 (20.0)
		Always	5 (26.3)	2 (13.3)	0 (0.0)	0 (0.0)
	12. Have you had difficulty doing your usual jobs because of problems with your teeth, mouth, or dentures?	Never	4 (21.1)	8 (53.3)	8 (42.1)	12 (80.0)
		Rarely	3 (15.8)	1 (6.7)	3 (15.8)	0 (0.0)
		Sometimes	6 (31.6)	1 (6.7)	6 (31.6)	3 (20.0)
		Recurrently	3 (15.8)	5 (33.3)	2 (10.5)	0 (0.0)
		Always	3 (15.8)	0 (0.0)	0 (0.0)	0 (0.0)
**Handicap**	13. Have you felt that life, in general, was less satisfying because of problems with your teeth, mouth, or dentures?	Never	8 (42.1)	7 (46.7)	15 (78.9)	14 (93.3)
		Rarely	2 (10.5)	2 (13.3)	2 (10.5)	0 (0.0)
		Sometimes	5 (26.3)	3 (20.0)	1 (5.3)	0 (0.0)
		Recurrently	2 (10.5)	2 (13.3)	1 (5.3)	1 (6.7)
		Always	2 (10.5)	1 (6.7)	0 (0.0)	0 (0.0)
	14. Have you been totally unable to function because of problems with your teeth, mouth, or dentures?	Never	5 (26.3)	8 (53.3)	10 (52.6)	13 (86.7)
		Rarely	5 (26.3)	1 (6.7)	4 (21.1)	0 (0.0)
		Sometimes	5 (26.3)	4 (26.7)	5 (26.3)	2 (13.3)
		Recurrently	2 (10.5)	2 (13.3)	0 (0.0)	0 (0.0)
		Always	2 (10.5)	0 (0.0)	0 (0.0)	0 (0.0)

It is observed that “physical pain”, “psychological discomfort”, “physical disability”, and “psychological disability” were the most reported aspects in the corresponding items in the questionnaire before the participants underwent the treatment.

The “physical pain” and “psychological discomfort” were the most reported domains by both G1 and G2 volunteers. Under “physical pain”, they reported discomfort eating because of the TMD, and under “psychological discomfort”, they reported being self-conscious. Regarding “physical disability”, G1 presented with the frequency of always having an unsatisfactory diet, as well as in “psychological disability”, with a higher frequency of difficulties relaxing because of the TMD.

In the post-treatment period, the answers improved in all the questions in both groups. However, “functional limitation” and “handicap” were the OHQOL aspects most referred to as having never been affected by the TMD.

In “functional limitation”, neither G1 nor G2 subjects had any more problems pronouncing a word; likewise, they did not feel the taste of food getting worse. In “handicap”, the volunteers reported no longer feeling that life, in general, had grown worse; similarly, they no longer felt hindered from carrying out their daily activities.

Table 2 shows the intragroup relationship in G1 and G2 – i.e., between the subjects of each group – regarding the severity of orofacial pain (with the VAS) and the perception of OHQOL before and after the intervention (with the OHIP-14 domains).

**Table 2:** Description of the participants regarding the median and intragroup relationship for the degree of orofacial pain, in the visual analog scale, and for the subjective perception of the oral health quality of life, in the OHIP-14 domains, before and after the treatment. João Pessoa, Paraíba, Brazil. 2020

	**G1**			**G2**		
	**BEFORE THE TREATMENT** **Median** **(Q_1_-Q_3_)**	**AFTER THE TREATMENT** **Median** **(Q_1_-Q_3_)**	p-value*	**BEFORE THE TREATMENT** **Median** **(Q_1_-Q_3_)**	**AFTER THE TREATMENT** **Median** **(Q_1_-Q_3_)**	**p-value** *
**Visual analog scale**	8 (7-10)	2 (0-5)	**0.001**	8 (5-9)	3 (0-5)	**0.002**
**Functional limitation**	1 (0-4)	0 (0-2)	0.180	0 (0-3)	0 (0-0)	**0.018**
**Physical pain**	6 (5-7)	2 (1-5)	**0.001**	6 (3-8)	1 (1-4)	**0.009**
**Psychological discomfort**	6 (4-8)	3 (1-5)	**0.001**	6 (2-8)	3 (0-4)	**0.007**
**Physical disability**	5 (4-7)	0 (0-3)	**0.001**	4 (2-6)	1 (0-3)	**0.003**
**Psychological disability**	3 (2-6)	1 (0-3)	**0.001**	2 (1-5)	2 (0-3)	0.070
**Social disability**	2 (2-5)	2 (0-5)	0.153	2 (0-3)	0 (0-6)	0.057
**Handicap**	2 (1-5)	0 (0-2)	**0.006**	1 (0-5)	0 (0-0)	**0.007**
**OHIP-14 total**	30 (23-35)	12 (3-22)	**0.001**	23 (14-31)	8 (2-15)	**0.002**

* Wilcoxon test

An improved relationship was verified in both the degree of orofacial pain (with the VAS) and the OHQOL (with the OHIP-14 total score) in both groups. Most domains in the questionnaire had this specific result, except for the “functional limitation” and “social disability” in G1 and “psychological disability” and “social disability” in G2.

In Table 3, the analysis after the therapy sessions verified a difference between the groups in only one of the domains in the protocol. The greatest difference between the groups after the individuals had been treated was perceived in the functional limitation.

**Table 3:** Intergroup relationship of the visual analog scale for the degree of orofacial pain and the subjective perception of the oral health quality of life in the OHIP-14 domains, before and after the treatment. João Pessoa, Paraíba, Brazil. 2020

	**G1 x G2**	**G1 x G2**
	**BEFORE THE TREATMENT**	**AFTER THE TREATMENT**
	p-value*	**p-value** *
**Visual analog scale**	0.336	0.607
**Functional limitation**	0.607	**0.036**
**Physical pain**	0.758	0.864
**Psychological discomfort**	0.430	0.784
**Physical disability**	0.157	0.891
**Psychological disability**	0.215	0.973
**Social disability**	0.319	0.137
**Handicap**	0.372	0.128
**OHIP-14 total**	0.157	0.632

* Mann-Whitney test

A correlation was observed between the VAS and total OHIP-14 score after the treatment in both G1 and G2, as shown in Table 4. Both correlations were strong and positive (i.e., the values increased or decreased proportionally in both groups).

**Table 4:** Correlation between age, the visual analog scale for the degree of orofacial pain, and the subjective perception of the oral health quality of life in the groups G1 and G2 before and after the treatment. João Pessoa, Paraíba, Brazil. 2020

	G1		G2	
	ρ	p-value*	ρ	p-value*
Age x	0.313	0.191	-0.090	0.749
VAS before the treatment				
Age x	-0.220	0.366	-0.114	0.686
VAS after the treatment				
Age x	0.159	0.515	-0.230	0.409
Total OHIP-14 before the treatment				
Age x	-0.348	0.144	-0.063	0.823
Total OHIP-14 after the treatment				
VAS before the treatment x	0.182	0.457	0.336	0.220
Total OHIP-14 before the treatment				
VAS after the treatment x	0.767	**0.000**	0.704	**0.003**
Total OHIP-14 after the treatment				

* Spearman correlation test

No correlation was found between the VAS and the OHIP-14 total score before the intervention, or between age and the VAS, and between age and the total OHIP-14 score before and after the treatment.

## DISCUSSION

Oral health is an essential part of the QOL of humans. This relationship is manifold and involves the physical, social, and psychological aspects^(23)^. Hence, the professionals must know and understand the oral/orofacial health and disease conditions, considering how the consequences in this region have harmful effects on the person’s overall well-being.

Having pain and discomfort is usually one of the most relevant positive and negative factors of the QOL^(23)^. It is particularly the case of orofacial pain, which is more significant for these individuals than other systemic conditions, such as diabetes, hypertension, and ulcer^(24)^. In this research, “physical pain”, “psychological discomfort”, “physical disability”, and “psychological disability” in the OHIP-14 were the most reported as “always” by the participants before the treatment.

This result was similar to another one published in the literature. Rodrigues and collaborators^(6)^ assessed the influence of TMD on the subjective perception of OHQOL using the OHIP-14. They verified that “physical pain”, “psychological discomfort”, and “psychological disability” were likewise the most reported domains by the sample investigated.

In another more recent study^(25)^, the researchers found an association between the pain and the QOL in five out of the seven domains of the same protocol: “psychological discomfort”, “physical disability”, “psychological disability”, “social disability”, and “handicap”. Thus, these data make evident these patients’ painful symptomatology, discomfort, and restrictions, with which they live in their daily activities when they do not have therapeutic care.

The present study revealed a strong positive correlation after the treatment in the improved degree of pain and self-perception of OHQOL in both G1 and G2. There was also a relationship of these aspects between the subjects of each group. This demonstrated that the individuals treated with OMT combined with active laser had positive responses, likewise those who received an intervention without the laser. It also indicates that the lower the degree of pain, the less impact on the OHQOL was perceived.

The diminished perception of pain in the two groups at the end of the treatment agrees with current research^(8)^, which also followed up and assessed the degree of pain with the VAS and the oral amplitude in people with the same disorder as those in this study. They were treated with OMT and photobiomodulation, using the same dosimetry parameters to apply LLL. The same improvement was perceived in those treated with OMT and active laser and with OMT and placebo laser.

Factors like stress, time of disease progression, severe loss of the vertical TMJ dimension^(26)^, the type of radiation applied, the tissue on which it is irradiated, and the immune status^(27)^ may influence the feedback of TMD patients when treated with photobiomodulation. Hence, these factors may explain the outcomes of using this treatment in the present study sample, as well as in the abovementioned current research. In both studies, the patients who had not been submitted to photobiomodulation also had the painful symptomatology diminished and the OHQOL improved.

No publication was found discussing the relationship between the self-perception of orofacial pain and the QOL in TMD patients submitted to the two said treatments. This highlights the importance of this paper to the scientific community, providing knowledge to the professionals who treat this population. Moreover, the fact that these study participants had their pain eased and their QOL proportionally restored demonstrates that they are directly related. Hence, they have effects not only on the body but also on the psychological status and social interaction^(8)^.

The OMT proved to be the treatment that most influenced the abovementioned findings. It aims at the functional recovery of the stomatognathic system, so the masticatory, swallowing, and speaking functions can be performed without pain, limitations, or risk of worsening the problem^(9)^. This was particularly verified in the present research, as seen in Table 3. At the end of the treatment, the OHIP-14 “functional limitation” domain was the only one with a statistically significant difference in the comparison between the groups – i.e., the functional recovery was the most perceived change in the OHQOL. This was further evidenced in G2, as seen in Table 2.

Therefore, the finding that these subjects belonged to the group treated with placebo laser stands out. The perception of 100% functional recovery to pronounce a word or taste the foods may have been influenced by this effect. Positive functional results, such as in mandibular mobility, have also been seen^(18)^, and another piece of research identified a greater decrease in mastication difficulties in subjects treated with orofacial myofunctional exercises combined with placebo laser^(17)^.

This evidence exists because the placebo response is a neurobiological event, whose activity in cortical areas has been associated with pain inhibition and the affective and cognitive center^(28)^. The patient’s expectations, a good patient-therapist relationship, and the sensation of receiving a more complete treatment with laser technology may explain this possibility^(29)^.

A piece of research was conducted with 11 women with muscular TMD, divided into two groups: An experimental group (EG), submitted to OMT combined with photobiomodulation, and a positive control group (CG), submitted to OMT combined with inactive photobiomodulation (placebo). The intervention was also made in 10 sessions, with 830-nm wavelength, furnishing a punctual dose of 3 J. Different from the findings in this study, the EG obtained increased measures of mandibular protrusion and opening movements and an improvement in the QOL assessment^(30)^. They demonstrated more expressive results when the sessions maintained the dose (3 J), unlike the criteria used in this research – which varied the doses, with 6 J from the first to the fifth session to ease the pain and 4 J beginning at the sixth session to biostimulate the functional gains obtained with the speech-language-hearing therapy.

This led to the reflection that, although the LLL triggers different action mechanisms in the organism and is positively reported in the interventions with combined treatment of TMD patients^(27)^, its clinical effectiveness occurs in different parameters, doses, and methodological criteria in the studies. Thus, the effects of photobiomodulation to optimize speech-language-hearing therapy need further evidence.

Given the above, this research reinforced the speech-language-hearing therapists’ follow-up of TMD patients with OMT, as they are the professionals responsible for this therapy, which is a reference in the field of oral-motor function. However, some limitations were identified in this study, such as the small sample size and the difference in the number of participants between the groups. This may have been one of the reasons why the research hypothesis was not answered – that people with muscular TMD submitted to photobiomodulation combined with OMT would have more gains in their QOL than those with the same characteristics submitted only to the OMT.

Thus, the scientific debate using this resource in speech-language-hearing therapy must continue, exploring the analyses with larger samples to obtain scientific evidence and consolidate the knowledge of the effects of photobiomodulation on the QOL of people with TMD.

## CONCLUSION

The people with TMD treated with photobiomodulation combined with OMT perceived an improvement in their OHQOL, as well as those treated with placebo laser. There was a strong positive correlation in both groups in the improvement of the degree of pain and self-perception of OHQOL.

## References

[1] De Leeuw R, Kasser G (2013). Orofacial Pain: Guidelines for assessment, diagnosis, and management..

[2] Tjakkes GH, Reinders JJ, Tenvergert EM, Stegenga B (2010). TMD pain: the effect on health related quality of life and the influence of pain duration. Health Qual Life Outcomes.

[3] de Souza Barbosa T, Gavião MB, Castelo PM, Leme MS (2016). Factors associated with oral health-related quality of life in children and preadolescents: a cross-sectional study. Oral Health Prev Dent.

[4] Rodrigues CA, Magri LV, Melchior MO, Mazzetto MO (2015). Avaliação do impacto na qualidade de vida de pacientes com disfunção temporomandibular. Rev Dor São Paulo.

[5] De Rossi SS, Greenberg MS, Liu F, Steinkeler A (2014). Temporomandibular disorders: evaluation and management. Med Clin North Am.

[6] Trize DM, Calabria MP, Franzolin SOB, Cunha CO, Marta SN (2018). A disfunção temporomandibular afeta a qualidade de vida?. Einstein (Sao Paulo).

[7] Paulino MR, Moreira VG, Lemos GA, Silva PLPD, Bonan PRF, Batista AUD (2018). Prevalência de sinais e sintomas de disfunção temporomandibular em estudantes pré-vestibulandos: associação de fatores emocionais, hábitos parafuncionais e impacto na qualidade de vida. Cien Saude Colet.

[8] Batista SL, Coêlho JF, Almeida LNA, Spinelli-Pessoa L, Vasconcelos ML, Alves GAS (2019). Amplitude oral e dor orofacial em pacientes com disfunção temporomandibular submetidos a laserterapia e a terapia miofuncional orofacial. Rev Bras Ciênc Saúde.

[9] de Felício CM, de Oliveira MM, da Silva MA (2010). Effects of orofacial myofunctional therapy on temporomandibular disorders. Cranio.

[10] Bianchini EMG (2001). Avaliação fonoaudiológica da motricidade oral: distúrbios miofuncionais orofaciais ou situações adaptativas. Dental Press Ortodon e Ortop Facial..

[11] Sassi FC, Silva AP, Santos RKS, Andrade CRF (2018). Tratamento para disfunções temporomandibulares: uma revisão sistemática. Audiol Commun Res.

[12] Maluf SA, Moreno BGD, Alfredo PP, Marques AP, Rodrigues G (2008). Exercícios terapêuticos nas desordens temporomandibulares: uma revisão de literatura. Fisioter Pesqui.

[13] Herpich CM, Amaral AP, Leal-Junior EC, Tosato JP, Gomes CA, Arruda ÉE (2015). Analysis of laser therapy and assessment methods in the rehabilitation of temporomandibular disorder: a systematic review of the literature. J Phys Ther Sci.

[14] Melchior MO, Machado BCZ, Magri LV, Mazzetto MO (2016). Efeito do tratamento fonoaudiológico após a laserterapia de baixa intensidade em pacientes com DTM: estudo descritivo. CoDAS.

[15] Matias AGC, Rocha AB, Santos CS, Fonseca MA (2014). Modulação da dor em portadores de disfunções temporomandibulares pela ação do laser – ASGAAL. Interscentia..

[16] Ahrari F, Madani AS, Ghafouri ZS, Tunér J (2014). The efficacy of low-level laser therapy for the treatment of myogenous temporomandibular joint disorder. Lasers Med Sci.

[17] Machado BCZ, Mazzetto MO, Da Silva MA, de Felício CM (2016). Effects of oral motor exercises and laser therapy on chronic temporomandibular disorders: a randomized study with follow-up. Lasers Med Sci.

[18] Chen J, Huang Z, Ge M, Gao M (2015). Efficacy of low-level laser therapy in the treatment of TMDs: a meta-analysis of 14 randomised controlled trials. J Oral Rehabil.

[19] Dworkin SF, Leresche L (1992). Research diagnostic criteria for temporomandibular disorders: review, criteria, examinations and specifications, critique. J Craniomandib Disord.

[20] Oliveira BH, Nadanovsky P (2005). Psychometric properties of the Brazilian version of the Oral Health Impact Profile-short form. Community Dent Oral Epidemiol.

[21] Gabardo MCL, Moysés ST, Moysés SJ (2013). Autopercepção de saúde bucal conforme o Perfil de Impacto da Saúde Bucal (OHIP) e fatores associados: revisão sistemática. Rev Panam Salud Publica.

[22] Dancey C, Reidy J. (2006). Estatística sem matemática para psicologia: usando SPSS para Windows..

[23] Carminatti M, Lavra-Pinto B, Franzon R, Rodrigues JA, Araújo FB, Gomes E (2017). Impacto da cárie dentária, maloclusão e hábitos orais na qualidade de vida relacionada à saúde oral em crianças pré-escolares. Audiol Commun Res.

[24] Kuroiwa DN, Marinelli JG, Rampani MS, Oliveira W, Nicodemo D (2011). Desordens temporomandibulares e dor orofacial: estudo da qualidade de vida medida pelo Medical Outcomes Study 36 – Item Short Form Health Survey. Rev Dor.

[25] Queiroz MF, Verli FD, Marinho SA, Paiva PCP, Santos SMC, Soares JA (2019). Dor, ansiedade e qualidade de vida relacionada à saúde bucal de pacientes atendidos no serviço de urgência odontológica. Cien Saude Colet.

[26] Catão MHCV, Oliveira PS, Costa RO, Carneiro VSM (2013). Avaliação da eficácia do laser de baixa intensidade no tratamento das disfunções têmporo-mandibular: estudo clínico randomizado. Rev CEFAC.

[27] Matos AS, Berretin-Felix G, Bandeira RN, Lima JAS, Almeida LNA, Alves GÂS (2018). Laser therapy applied to orofacial motricity: perception of members of the Brazilian Orofacial Motricity Association - Abramo. Rev CEFAC.

[28] George SZ, Robinson ME (2010). Dynamic nature of the placebo response. J Orthop Sports Phys Ther.

[29] Kisaalita NR, Robinson ME (2012). Analgesic placebo treatment perceptions: acceptability, efficacy, and knowledge. J Pain.

[30] Alves GAS, Gondim YRDR, Lima JAS, Silva MAPD, Florêncio DSF, Almeida LNA (2021). Effects of photobiomodulation associated with orofacial myofactional therapy on temporomandibular joint dysfunction. CoDAS.

