# Supplementary material for: Effects of photobiomodulation combined with orofacial myofunctional therapy on the quality of life of individuals with temporomandibular disorder
Source: Codas. 2022 Apr 8;34(5):e20200313. doi: 10.1590/2317-1782/20212020313 (PMC9886175; doi:10.1590/2317-1782/20212020313)
Supplement: Translation

# Translation: Efeitos da fotobiomodulação associada a terapia miofuncional orofacial na qualidade de vida de indivíduos com disfunção temporomandibular

**Authors:** Wellyda Cinthya Félix Gomes da Silva Dias, Renata Veiga Andersen Cavalcanti, Hipólito Virgílio Magalhães, Leandro de Araújo Pernambuco, Giorvan Ânderson dos Santos Alves

**Affiliations:** 1 Programa Associado de Pós-graduação em Fonoaudiologia, Universidade Federal da Paraíba – UFPB - João Pessoa (PB), Brasil.; 2 Programa Associado de Pós-graduação em Fonoaudiologia, Universidade Federal do Rio Grande do Norte - UFRN - Natal (RN), Brasil.

**Keywords:** Transtornos da Articulação Temporomandibular, Qualidade de Vida, Terapia a Laser, Terapia Miofuncional, Fonoaudiologia

## Abstract

**Objetivo:**

Analisar o impacto na qualidade de vida relacionada à saúde oral de indivíduos com disfunção temporomandibular, antes e após o tratamento de fotobiomodulação associada a terapia miofuncional orofacial.

**Método:**

Estudo do tipo ensaio clínico randomizado, controlado e cego, com 34 voluntários distribuídos aleatoriamente em dois grupos: G1, que recebeu a Terapia Miofuncional Orofacial (TMO) associada a fotobiomodulação e o G2, tratado pela TMO associada ao laser inativo. Os indivíduos foram submetidos, primeiramente, à avaliação para serem classificados de acordo com o grau da dor orofacial pela Escala Visual Analógica (EVA) e também quanto ao impacto da qualidade de vida relacionada a saúde oral (QVRSO) pelo questionário *Oral Health Impact Profile – Short form* (OHIP-14). Os dados obtidos foram analisados estatisticamente. Foi adotado o nível de significância de 0,05 (95%).

**Resultados:**

“Dor física”, “desconforto psicológico”, “limitação física” e “limitação psicológica” foram os aspectos mais impactantes na QVRSO. O G1 apresentou respostas positivas para o respectivo tratamento, assim como o G2. Observou-se correlação positiva e de grau forte para EVA e escore total do OHIP-14 em ambos os grupos após tratamento. Porém, os indivíduos do grupo controle (G2) evidenciaram que a recuperação funcional foi o aspecto que mais se percebeu de mudança positiva na QVRSO em comparação ao grupo experimental (G1).

**Conclusão:**

As pessoas que receberam fotobiomodulação associada a TMO-perceberam melhora da QVRSO, assim como as tratadas com o laser placebo. Houve correlação positiva e forte em ambos os grupos na melhora do grau da dor e autopercepção da QVRSO.

## INTRODUÇÃO

A Disfunção Temporomandibular (DTM) é conceituada pela *American Academy of Orofacial Pain* (AAOP) como uma série de condições clínicas envolvendo os músculos mastigatórios, a articulação temporomandibular (ATM) e as estruturas do sistema estomatognático associadas^(1)^.

É evidenciada por diferentes sinais e sintomas, sendo a dor a principal característica referida pelos sujeitos acometidos por essa afecção e em algumas vezes o maior motivo para se buscar tratamento^(2)^. Por isso, a DTM é considerada a maior causa de dor não dentária na região orofacial^(3)^. A dor pode ser manifestada na região da ATM, face, cabeça, pescoço e ouvidos; durante a abertura e fechamento da mandíbula; além de haver limitação nos movimentos mandibulares, ruídos articulares e dificuldades na fala, mastigação e deglutição^(4)^. Além disso, é comum haver relatos do aumento da dor/desconforto durante as atividades destas funções^(5)^.

Logo, há influência direta e negativa destes problemas sob a saúde física e mental dos pacientes, afetando suas atividades escolares, profissionais e sociais; levando até mesmo a um desequilíbrio afetivo e cognitivo^(6)^. Sendo assim, ocorre repercussão na qualidade de vida (QV) dessas pessoas de forma danosa e, sobretudo, na qualidade de vida relacionada com a saúde oral (QVRSO) com maior comprometimento a depender da gravidade da DTM^(7)^.

Esse fato reforça o saber de que o atendimento de indivíduos com DTM demanda um olhar clínico ampliado por envolver diversos aspectos e, em especial a QV, pois está associada à percepção subjetiva que os mesmos têm sobre sua posição de vida; no contexto de valores e cultura nos quais eles vivem e em relação a seus objetivos, expectativas e preocupações – o que torna a investigação do grau de comprometimento da QV muito importante para os profissionais de saúde que os tratam^(4)^.

Porém, em muitos casos, além da percepção e os sentimentos de pacientes em relação à saúde oral serem ignorados, na literatura há poucos estudos que analisam o impacto das condições dolorosas da DTM sobre a QV, quando comparados ao número de publicações sobre diagnóstico, sinais e sintomas da DTM^(8)^.

Diante desse contexto, são propostas diversas abordagens terapêuticas para os quadros clínicos de DTM que visam amenizar e tratar tais consequências, além de trazer mais conforto ao paciente^(4)^. Dentre elas, se destaca a atuação do fonoaudiólogo na área de motricidade orofacial, por meio da Terapia Miofuncional Orofacial (TMO). Esta tem por finalidade promover estabilidade miofuncional orofacial^(9)^, ao modificar os padrões musculares e funcionais^(10)^, permitindo a redução da dor^(11)^; adequação da amplitude de movimentos mandibulares^(12)^; e consequentemente a readequação das funções de respiração, mastigação, deglutição e fala^(10)^.

O uso do Laser de Baixa Intensidade (LBI) ou Fotobiomodulação nas DTMs também tem sido descrito e discutido nas pesquisas científicas, por se caracterizar como uma intervenção não farmacêutica, não invasiva, rápida e segura ao paciente devido a reações favoráveis nas dores miogênicas e articulares, sob os efeitos analgésicos, anti-inflamatório e biomodulador das funções fisiológicas celulares^(13,14)^.

Autores apontam que estes efeitos se caracterizam como positivos na redução da dor^(15)^; aumento da amplitude de abertura bucal^(16)^; nos sinais e sintomas e recuperação funcional^(17)^. No entanto, alguns estudos de meta-análises^(13,18)^ demonstraram que os grupos com laser ativo tiveram redução da dor semelhantes aos de grupos placebos, embora o primeiro apresentado resultados melhores no quadro funcional dos indivíduos^(18)^.

Pesquisadores^(17)^ reforçam ainda que combinações de tratamentos para controle álgico e treino das funções motoras orofaciais são sugestivas e favoráveis na reabilitação das DTMs. Sendo assim, o objetivo deste estudo é analisar o impacto na qualidade de vida relacionada à saúde oral de indivíduos com disfunção temporomandibular, antes e após o tratamento de fotobiomodulação associada a terapia miofuncional orofacial, bem como verificar possível correlação entre o grau de dor orofacial e a autopercepção desse impacto.

## MÉTODO

Trata-se de um estudo de intervenção terapêutica, longitudinal e do tipo ensaio clínico randomizado, controlado e cego. Foi aprovado pelo Comitê de Ética e Pesquisa do Centro de Ciências da Saúde da Universidade Federal da Paraíba, sob o parecer de número 3.354.075, conforme os princípios éticos da Resolução do Conselho Nacional de Saúde – CNS de nº 466/2012.

A pesquisa foi realizada na Clínica Escola de Fonoaudiologia da UFPB, envolvendo participantes de ambos os sexos e a amostra foi constituída pelo total de 34 voluntários que buscaram os serviços de Fonoaudiologia por livre demanda.

Por meio da realização de triagens, foram considerados como critérios de elegibilidade a idade entre 18 e 59 anos; os sujeitos que apresentassem dor orofacial caracterizada como DTM Muscular, de acordo com a aplicação do protocolo *Research Diagnostic Criteria for Temporomandibular Disorders* – RDC/TMD – Eixo I^(19)^; os que tivessem disponibilidade de horário para a realização do tratamento e que concordassem participar do estudo assinando o Termo de Consentimento Livre e Esclarecido (TCLE). Foram excluídos aqueles que referissem histórico de comprometimento neurológico; malformações, tumores, traumas ou cirurgias na região da face e pescoço; uso de prótese dentária ou aparelho ortodôntico; os que já tivessem realizado ou que estivessem em terapia fonoaudiológica; como também os que fizessem uso de medicação miorrelaxante e/ou anti-inflamatória ou que tenham o feito por mais de um ano.

Além destes fatores, também foram considerados os critérios que contraindicam a realização da fotobiomodulação: pacientes gestantes; com fotos sensibilidade; com glaucoma; que tivessem marcapasso ou outro implante eletrônico e/ou tecido infeccionado próximo ao local da irradiação.

A coleta dos dados da pesquisa foi precedida pela randomização dos participantes de modo simples em grupos, por meio de sorteio. Esta distribuição aleatória foi destinada aos grupos: G1 – Grupo Experimental que recebeu o tratamento de Terapia Miofuncional Orofacial (TMO) associada a fotobiomodulação; e o G2 – Grupo Controle Positivo, que recebeu a TMO associada ao laser inativo (placebo).

Os integrantes do estudo passaram por uma avaliação antes e após o período de intervenção terapêutica, realizando os seguintes procedimentos: Avaliação do impacto da qualidade de vida relacionada à saúde oral (QVRSO) pelo questionário *Oral Health Impact Profile – Short form* (OHIP-14) e também do grau da dor orofacial por meio da Escala Visual Analógica (EVA).

A qualidade de vida relacionada com a saúde oral foi analisada por meio do questionário *Oral Health Impact Profile – Short form* (OHIP-14) – desenvolvido por Slade (1997), em sua forma já traduzida e validada para o português do Brasil^(20)^. Foi aplicado em forma de entrevista, levando em conta a percepção subjetiva do paciente sobre o seu problema nos últimos seis meses.

O instrumento possui 14 perguntas, sendo duas para cada um dos seus sete domínios (limitação funcional, dor física, desconforto psicológico, limitação física, limitação psicológica, limitação social e incapacidade). Para cada questão, há cinco opções de respostas: nunca, raramente, às vezes, repetidamente e sempre. Conforme o método de adição^(21)^, estas são graduadas, respectivamente, como zero, um, dois, três e quatro pontos. A pontuação de cada um dos domínios pode variar de 0 a 8 pontos, com maiores escores significando maior comprometimento. Já para um escore total do OHIP-14, as respostas ordinais somadas podem variar de 0 a 56 pontos – significando maior impacto negativo na saúde oral.

A EVA foi aplicada considerando uma linha não numerada de 10 cm. A extremidade do lado esquerdo significava afirmar “nenhuma dor” e a do lado direito a “pior dor possível”, ou seja, quanto mais essa marcação fosse próxima da extremidade esquerda, melhor seria a classificação para o grau de dor e quanto mais perto da direita, pior seria esta classificação.

Os voluntários foram informados sobre o preenchimento da escala, e em seguida fizeram a autoanálise da dor espontânea, marcando um ponto na linha onde melhor representava o grau de sua dor no momento da avaliação. Para a análise do resultado, o avaliador usava uma régua sob a linha para identificar a numeração apontada entre 0 e 10 e transcrevia este resultado para a ficha de evolução clínica do paciente.

A intervenção consistiu em encontros semanais, com um total de 13 sessões. Uma destinada para a avaliação e aplicação dos instrumentos citados; uma exclusiva para orientações relacionadas a esclarecimentos das condições clínicas, hábitos prejudiciais que desencadeiam as dores e instruções de como estas podiam ser aliviadas em casa; 10 sessões para a terapia propriamente dita e uma outra específica para a reavaliação. Cada atendimento de fonoterapia teve duração total de 45 minutos, sendo 30 minutos destinada à TMO, bem como 15 e 12 minutos na 1ª e 2ª fase, respectivamente, para a aplicação do LBI. A equipe foi formada por 4 pesquisadores calibrados, com formação em Fotobiomodulação, e com experiência de 3 anos atuando nos procedimentos necessários.

O protocolo da TMO^(9)^ para a fonoterapia foi o mesmo para os dois grupos, compreendendo o uso de estratégias como a termoterapia e massoterapia com massagens lentas e profundas para favorecer a circulação sanguínea local e aliviar a dor; mioterapia – constituindo de exercícios isométricos e isotônicos para grupos musculares específicos (lábios, língua, bochechas e músculos mandibulares), visando a melhora da flexibilidade, coordenação, simetria e estabilização dos movimentos funcionais das ATMs; e também realizado o treino com as funções orofaciais, ou seja, utilizando a função de mastigação como exercício e treino.

A LBI ativa correspondeu à aplicação bilateral do laser de baixa intensidade de Arsenieto de Gálio-Alumínio (AsGal), equipamento Laser Pulse Line da marca IBRAMED. O protocolo adotado – elaborado a partir das discussões no Núcleo de Estudos em Linguagem e Funções Estomatognáticas NELF, consistiu no modo de aplicação pontual, em cinco locais na região da ATM: polo lateral; pontos superior, anterior, posterior e inferior da posição condilar; e nos locais dolorosos dos músculos masseter, temporal, esternocleidomastóideo e trapézio, relatados pelos indivíduos.

Com comprimento de onda de 830 nm (infravermelho) e irradiação com dois objetivos distintos em fases: a primeira (1ª à 5ª sessão) com dosimetria de 6J e fluência de 51 J/cm^2^, com a finalidade de analgesia do quadro doloroso e a segunda (a partir da 6ª sessão) com a dosimetria de 4J e fluência de 34 J/cm^2^, para bioestimular os ganhos funcionais promovidos pela terapia fonoaudiológica. Já a LBI inativa ocorreu de forma simulada proporcionando o efeito placebo.

Os dados obtidos foram tabulados e armazenados em planilha do *Microsoft Excel* 2013 para serem gerenciados. Realizou-se análise estatística tanto descritiva como inferencial utilizando o software *Statistical Package for the Social Sciences* (*SPSS) for Windows*, versão 21.0. Na descritiva, das variáveis qualitativas, foram aplicadas as frequências absoluta e relativa (percentual); e para as variáveis quantitativas, usada a média e mediana como medidas de tendência central, bem como desvio padrão e os quartis 25 e 75 como medidas de variabilidade.

Já na inferencial, para analisar a relação intragrupo das variáveis quantitativas e qualitativas, utilizou-se o teste de Wilcoxon e, para a relação entre os grupos, o teste de Mann-Whitney. Para verificar a correlação entre os dados quantitativos, foi aplicado o teste de Correlação de Spearman, o qual obtém resultados em percentual e pode-se ter valores positivos e negativos. O grau de correlação foi considerado fraco quando o valor resultou até 0,3; moderado entre 0,3 e 0,7 e forte quando encontrado valor acima de 0,7^(22)^. Neste estudo foi adotado o nível de significância de 0,05 (95%).

## RESULTADOS

No presente estudo, foram avaliados e tratados 34 indivíduos com diagnóstico de DTM muscular, sendo no G1 16 (84,2%) do sexo feminino e 3 (15,8%) do sexo masculino, com média de idade de 32,16±8,60 anos. Já no G2, 11 (73,3%) do sexo feminino e 4 (26,7%) do sexo masculino, com média de idade de 34,67±13,05 anos. A caracterização da amostra de acordo com a percepção subjetiva da qualidade de vida relacionada com a saúde oral pré e pós tratamento, para cada questão do OHIP-14, está demonstrada na Tabela 1.

**Tabela 1:** Distribuição absoluta e relativa dos participantes quanto a percepção subjetiva da qualidade de vida relacionada com a saúde oral pré e pós tratamento. João Pessoa, PB. 2020

**DOMÍNIOS DO OHIP-14**	**PERGUNTAS**	**RESPOSTAS**	**PRÉ TRATAMENTO**		**PÓS TRATAMENTO**	
			**G1**	**G2**	**G1**	**G2**
			**n (%)**	**n (%)**	**n (%)**	**n (%)**
**Limitação funcional**	1. Você teve problemas para falar alguma palavra?	Nunca	10 (52,6)	10 (66,7)	12 (63,2)	15 (100,0)
		Raramente	1 (5,3)	1 (6,7)	1 (5,3)	0 (0,00)
		Às vezes	4 (21,1)	3 (20,0)	6 (31,6)	0 (0,0)
		Repetidamente	2 (10,5)	1 (6,7)	0 (0,0)	0 (0,0)
		Sempre	2 (10,5)	0 (0,0)	0 (0,0)	0 (0,0)
	2. Você sentiu que o sabor dos alimentos tem piorado?	Nunca	14 (73,7)	9 (60,0)	15 (78,9)	15 (100,0)
		Raramente	1 (5,3)	2 (13,3)	3 (15,8)	0 (0,0)
		Às vezes	2 (10,5)	3 (20,0)	1 (5,3)	0 (0,0)
		Repetidamente	0 (0,0)	1 (6,7)	0 (0,0)	0 (0,0)
		Sempre	2 (10,5)	0 (0,0)	0 (0,0)	0 (0,0)
**Dor física**	3. Você sentiu dores fortes em sua boca?	Nunca	2 (10,5)	0 (0,0)	7 (36,8)	7 (46,7)
	3.	Raramente	0 (0,0)	3 (20,0)	4 (21,1)	2 (13,3)
	3.	Às vezes	6 (31,6)	3 (20,0)	4 (21,1)	3 (20,0)
	3.	Repetidamente	5 (26,3)	5 (33,3)	4 (21,1)	2 (13,3)
	3.	Sempre	6 (31,6)	4 (26,7)	0 (0,0)	1 (5,9)
	4. Você tem se sentido incomodado ao comer algum alimento?	Nunca	0 (0,0)	3 (20,0)	7 (36,8)	6 (40,0)
	3.	Raramente	3 (15,8)	1 (6,7)	3 (15,8)	3 (20,0)
	3.	Às vezes	1 (5,3)	1 (6,7)	5 (26,3)	3 (20,0)
	3.	Repetidamente	6 (31,6)	3 (20,0)	3 (15,8)	1 (6,7)
	3.	Sempre	9 (47,4)	7 (46,7)	1 (5,3)	2 (13,3)
**Desconforto psicológico**	5. Você ficou preocupado (a)?	Nunca	1 (5,3)	0 (0,00)	6 (31,6)	6 (40,0)
		Raramente	1 (5,3)	2 (13,3)	6 (31,6)	1 (6,7)
		Às vezes	5 (26,3)	4 (26,7)	3 (15,8)	6 (40,0)
		Repetidamente	3 (15,8)	3 (20,0)	2 (10,5)	2 (13,3)
		Sempre	9 (47,4)	6 (40,0)	2 (10,5)	0 (0,0)
	6. Você se sentiu estressado (a)?	Nunca	0 (0,0)	3 (20,0)	5 (26,3)	5 (33,3)
		Raramente	0 (0,0)	1 (6,7)	4 (21,1)	3 (20,0)
		Às vezes	6 (31,6)	2 (13,3)	4 (21,1)	3 (20,0)
		Repetidamente	4 (21,1)	5 (33,3)	3 (15,8)	2 (13,3)
		Sempre	9 (47,4)	4 (26,7)	3 (15,8)	2 (13,3)
**Limitação física**	7. Sua alimentação ficou prejudicada?	Nunca	2 (10,5)	2 (13,3)	11 (57,9)	8 (53,3)
		Raramente	0 (0,0)	0 (0,0)	2 (10,5)	0 (0,0)
		Às vezes	5 (26,3)	6 (40,0)	4 (21,1)	7 (46,7)
		Repetidamente	5 (26,3)	4 (26,7)	2 (10,5)	0 (0,0)
		Sempre	7 (36,8)	3 (20,0)	0 (0,0)	0 (0,0)
	8. Você teve que parar suas refeições?	Nunca	3 (15,8)	5 (33,3)	10 (52,6)	10 (66,7)
		Raramente	3 (15,8)	4 (26,7)	7 (36,8)	2 (13,3)
		Às vezes	6 (31,6)	2 (13,3)	2 (10,5)	3 (20,00)
		Repetidamente	4 (21,1)	3 (20,0)	0 (0,0)	0 (0,0)
		Sempre	3 (15,8)	1 (6,7)	0 (0,0)	0 (0,0)
**Limitação psicológica**	9. Você encontrou dificuldade para relaxar?	Nunca	1 (5,3)	2 (13,3)	5 (26,3)	5 (33,3)
	1.	Raramente	1 (5,3)	3 (20,0)	5 (26,3)	2 (13,3)
	1.	Às vezes	4 (21,1)	6 (40,0)	5 (26,3)	4 (26,7)
	1.	Repetidamente	6 (31,6)	1 (6,7)	4 (21,1)	1 (6,7)
	1.	Sempre	7 (36,8)	3 (20,0)	0 (0,0)	3 (20,0)
	10. Você se sentiu envergonhado (a)?	Nunca	10 (52,6)	8 (53,3)	12 (63,2)	13 (86,7)
		Raramente	1 (5,3)	1 (6,7)	6 (23,6)	0 (0,0)
		Às vezes	5 (26,3)	3 (20,0)	1 (5,3)	2 (13,3)
		Repetidamente	0 (0,0)	2 (13,3)	0 (0,0)	0 (0,0)
		Sempre	3 (15,8)	1 (6,7)	0 (0,0)	0 (0,0)
**Limitação social**	11. Você ficou irritado (a) com outras pessoas?	Nunca	9 (47,4)	7 (46,7)	8 (42,1)	10 (66,7)
		Raramente	2 (10,5)	2 (13,3)	3 (15,8)	1 (6,7)
		Às vezes	2 (10,5)	0 (0,0)	4 (21,1)	1 (6,7)
		Repetidamente	1 (5,3)	4 (26,7)	4 (21,1)	3 (20,0)
		Sempre	5 (26,3)	2 (13,3)	0 (0,0)	0 (0,0)
	12. Você teve dificuldade para realizar suas atividades diárias?	Nunca	4 (21,1)	8 (53,3)	8 (42,1)	12 (80,0)
		Raramente	3 (15,8)	1 (6,7)	3 (15,8)	0 (0,0)
		Às vezes	6 (31,6)	1 (6,7)	6 (31,6)	3 (20,0)
		Repetidamente	3 (15,8)	5 (33,3)	2 (10,5)	0 (0,0)
		Sempre	3 (15,8)	0 (0,0)	0 (0,0)	0 (0,0)
**Incapacidade**	13. Você sentiu que a vida, em geral, ficou pior?	Nunca	8 (42,1)	7 (46,7)	15 (78,9)	14 (93,3)
		Raramente	2 (10,5)	2 (13,3)	2 (10,5)	0 (0,0)
		Às vezes	5 (26,3)	3 (20,0)	1 (5,3)	0 (0,0)
		Repetidamente	2 (10,5)	2 (13,3)	1 (5,3)	1 (6,7)
		Sempre	2 (10,5)	1 (6,7)	0 (0,0)	0 (0,0)
	14. Você tem estado sem poder fazer suas atividades diárias?	Nunca	5 (26,3)	8 (53,3)	10 (52,6)	13 (86,7)
		Raramente	5 (26,3)	1 (6,7)	4 (21,1)	0 (0,0)
		Às vezes	5 (26,3)	4 (26,7)	5 (26,3)	2 (13,3)
		Repetidamente	2 (10,5)	2 (13,3)	0 (0,0)	0 (0,0)
		Sempre	2 (10,5)	0 (0,0)	0 (0,0)	0 (0,0)

Observa-se que a “dor física”, “desconforto psicológico”, “limitação física” e “limitação psicológica” foram os aspectos do questionário mais relatados pelas suas questões correspondentes antes dos participantes se submeterem ao tratamento.

A “dor física” e “desconforto psicológico” foram os domínios mais frequentemente referidos pelos voluntários tanto em G1 quanto em G2. Na “dor física”, em relação ao incômodo em comer algum alimento por causa da DTM e em quanto ao “desconforto psicológico”, para a sensação de preocupação. No que se refere à “limitação física”, o G1 foi quem apresentou a frequência de sempre ter prejuízos na alimentação, assim como no quesito “limitação psicológica” – com maior frequência em ter encontrado dificuldade para relaxar em decorrência da DTM.

No período pós-tratamento, foi percebida melhora das respostas para todas as perguntas em ambos os grupos. Porém, “limitação funcional” e “incapacidade” foram os aspectos da qualidade vida relacionados com a saúde oral mais frequentemente respondidos como nunca afetados por influência da DTM.

Em “limitação funcional”, os sujeitos tanto em G1 quanto em G2 não mais tiveram problemas para falar alguma palavra; bem como não mais sentiram piora no sabor dos alimentos. Em “incapacidade”, os voluntários relataram não mais sentirem que a vida, em geral, piorou; da mesma maneira que não mais se sentiram impedidos de realizarem suas atividades diárias.

A Tabela 2 ilustra a relação intragrupo de G1 e G2, ou seja, entre os sujeitos de cada grupo, sobre a gravidade da dor orofacial pela EVA e percepção da qualidade de vida relacionada com a saúde oral antes e após a intervenção, pelos domínios do OHIP-14.

**Tabela 2:** Descrição dos participantes quanto à mediana e relação intragrupos da escala visual analógica para o grau da dor orofacial e percepção subjetiva da qualidade de vida relacionada com a saúde oral pelos domínios do OHIP-14 pré e pós tratamento. João Pessoa, PB. 2020

	**G1**			**G2**		
	**PRÉ TRATAMENTO** **Mediana** **(Q25-Q75)**	**PÓS TRATAMENTO** **Mediana** **(Q25-Q75)**	p-valor*	**PRÉ TRATAMENTO** **Mediana** **(Q25-Q75)**	**PÓS TRATAMENTO** **Mediana** **(Q25-Q75)**	**p-valor** *
**Escala visual analógica**	8 (7-10)	2 (0-5)	**0,001**	8 (5-9)	3 (0-5)	**0,002**
**Limitação funcional**	1 (0-4)	0 (0-2)	0,180	0 (0-3)	0 (0-0)	**0,018**
**Dor física**	6 (5-7)	2 (1-5)	**0,001**	6 (3-8)	1 (1-4)	**0,009**
**Desconforto psicológico**	6 (4-8)	3 (1-5)	**0,001**	6 (2-8)	3 (0-4)	**0,007**
**Limitação física**	5 (4-7)	0 (0-3)	**0,001**	4 (2-6)	1 (0-3)	**0,003**
**Limitação psicológica**	3 (2-6)	1 (0-3)	**0,001**	2 (1-5)	2 (0-3)	0,070
**Limitação social**	2 (2-5)	2 (0-5)	0,153	2 (0-3)	0 (0-6)	0,057
**Incapacidade**	2 (1-5)	0 (0-2)	**0,006**	1 (0-5)	0 (0-0)	**0,007**
**OHIP-14 total**	30 (23-35)	12 (3-22)	**0,001**	23 (14-31)	8 (2-15)	**0,002**

* Teste de Wilcoxon

Verifica-se que houve relação da melhora tanto no grau da dor orofacial pela EVA, quanto para a QVRSO pelo escore total do OHIP-14 em ambos os grupos. A maioria dos respectivos domínios do questionário especificaram esses resultados, com exceção dos domínios “limitação funcional” e “limitação social” entre o G1 e dos domínios “limitação psicológica” e “limitação social” entre o G2.

Já na Tabela 3, a análise entre os grupos após as sessões terapêuticas constatou que apenas um dos domínios do protocolo apresentou diferença entre eles. A limitação funcional indicou ter sido o domínio que foi mais percebida a diferença entre os grupos depois que os indivíduos foram tratados.

**Tabela 3:** Relação intergrupos da escala visual analógica para o grau da dor orofacial e percepção subjetiva da qualidade de vida relacionada com a saúde oral pelos domínios do OHIP-14 pré e pós tratamento. João Pessoa, PB. 2020

	**G1 x G2**	**G1 x G2**
	**PRÉ TRATAMENTO**	**PÓS TRATAMENTO**
	p-valor*	**p-valor** *
**Escala visual analógica**	0,336	0,607
**Limitação funcional**	0,607	**0,036**
**Dor física**	0,758	0,864
**Desconforto psicológico**	0,430	0,784
**Limitação física**	0,157	0,891
**Limitação psicológica**	0,215	0,973
**Limitação social**	0,319	0,137
**Incapacidade**	0,372	0,128
**OHIP-14 total**	0,157	0,632

* Teste de Mann-Whitney

Na Tabela 4, foi observada correlação entre EVA e escore total do OHIP-14 após o tratamento tanto em G1 quanto em G2. Ambas correlações foram positivas – ou seja, os valores aumentaram ou diminuíram proporcionalmente nos dois grupos – e de grau forte.

**Tabela 4:** Correlação entre idade, escala visual analógica para o grau da dor orofacial e percepção subjetiva da qualidade de vida relacionada com a saúde oral nos grupos G1 e G2 pré e pós tratamento. João Pessoa, PB. 2020

	G1		G2	
	ρ	p-valor*	ρ	p-valor*
Idade x	0,313	0,191	-0,09	0,749
EVA pré tratamento				
Idade x	-0,22	0,366	-0,114	0,686
EVA pós tratamento				
Idade x	0,159	0,515	-0,23	0,409
OHIP-14 total pré tratamento				
Idade x	-0,348	0,144	-0,063	0,823
OHIP-14 total pós tratamento				
EVA pré tratamento x	0,182	0,457	0,336	0,22
OHIP-14 total pré tratamento				
EVA pós tratamento x	0,767	**0**	0,704	**0,003**
OHIP-14 total pós tratamento				

* Teste de Correlação de Spearman

Não houve correlação entre EVA e escore total do OHIP-14 antes da intervenção, como também entre idade e EVA; idade e escore total do OHIP-14 no pré e pós tratamento.

## DISCUSSÃO

A saúde oral integra e é essencial para a qualidade de vida dos seres humanos. Esta relação se configura sob variadas formas envolvendo os aspectos físico, social e psicológico^(23)^. Por isto, vê-se a importância de os profissionais conhecerem e compreenderem as condições de saúde e doença oral/orofacial – considerando os efeitos nocivos das repercussões nesta região para o bem-estar global de uma pessoa.

A ocorrência de dor e desconforto costuma ser considerada como um dos fatores positivos e negativos mais relevantes para a qualidade de vida^(23)^. Em especial, a dor orofacial, que tem sido mais marcante para os indivíduos do que outras condições sistêmicas – como a diabetes, hipertensão e úlcera^(24)^. Nesta pesquisa, os itens “dor física”, “desconforto psicológico”, “limitação física” e “limitação psicológica” do OHIP-14 foram os mais sempre referidos pelos participantes antes de serem tratados.

Este resultado foi semelhante a um publicado na literatura. Rodrigues e colaboradores^(6)^, ao avaliarem a influência da DTM na percepção subjetiva da QVRSO aplicando o OHIP-14, verificaram que os aspectos “dor física”, “desconforto psicológico” e “limitação psicológica” também foram os mais frequentemente apontados pela amostra investigada.

E, em um outro estudo mais recente^(25)^, os pesquisadores encontraram associação entre dor e qualidade de vida em 5 dos 7 domínios do mesmo protocolo: “desconforto psicológico”, “limitação física”, “limitação psicológica”, “limitação social” e em “incapacidade”. Dessa forma, tais dados evidenciam a notoriedade de sintomatologia dolorosa, incômodo e restrições que esses pacientes possuem e convivem em suas atividades cotidianas na ausência de cuidados terapêuticos.

Com este estudo, foi possível constatar que após o tratamento houve correlação positiva e forte de melhora para o grau da dor e autopercepção da QVRSO tanto em G1 quanto em G2; bem como relação destes mesmos aspectos entre os sujeitos de cada grupo. Isto demonstrou que os indivíduos tratados com a TMO associada ao laser ativo apresentaram respostas positivas igualmente aos que receberam a intervenção sem o laser, além de indicar também que quanto menor o grau da dor, menor foi o impacto percebido na QVRSO.

Quanto à redução da percepção da dor nos dois grupos ao fim do tratamento, foi vista concordância com uma pesquisa atual^(8)^, que também acompanhou e avaliou o grau da dor pela EVA e a amplitude oral em pessoas portadoras da mesma disfunção que a deste estudo, tratando-as com TMO e fotobiomodulação – e sob os mesmos parâmetros de dosimetria ao aplicar o laser de baixa intensidade. Houve igual percepção de melhora tanto nas pessoas tratadas com TMO e o laser ativo, quanto nas que receberam a TMO e o laser placebo.

Fatores como estresse, tempo de progressão da doença, perda severa da dimensão vertical na ATM^(26)^, tipo de radiação aplicada, tecido que está sendo irradiado, como também as condições imunológicas^(27)^ são possíveis aspectos que podem influenciar no feedback do paciente que possui DTM frente à fotobiomodulação como tratamento. Logo, tais fatores podem explicar as repercussões do uso desse tratamento na amostra do presente trabalho, bem como na pesquisa recente citada acima, visto que os pacientes – em ambos estudos – que não foram submetidos à fotobiomodulação também apresentaram diminuição da sintomatologia dolorosa e melhora da QVRSO.

Não foram encontradas publicações que discutissem a relação da autopercepção da dor orofacial e qualidade de vida em pacientes com DTM submetidos a ambos tratamentos mencionados – o que torna este trabalho importante para comunidade científica e de conhecimento aos profissionais que atendem esta população. Contudo, o fato de os participantes deste estudo terem sentido alívio da dor e, proporcionalmente, o restabelecimento da qualidade de vida, ratifica que ambos são diretamente relacionados, uma vez que os mesmos geram efeitos não só ao corpo, mas também ao estado psicológico e convivência social^(8)^.

A terapia miofuncional orofacial demonstrou ter sido o tratamento mais influente para os achados acima referidos. Ela tem por finalidade a recuperação funcional do sistema estomatognático, de modo que as funções de mastigar, deglutir e falar possam ser realizadas sem dor e limitações ou risco de agravar o problema^(9)^– o que pôde ser, especialmente, conferido também na presente pesquisa, como visto na Tabela 3. Ao término do tratamento, o domínio “limitação funcional” do OHIP-14 foi o único que demonstrou diferença estatisticamente significante quando os grupos foram comparados um com o outro, ou seja, a recuperação funcional foi o que mais se percebeu de mudança na QVRSO – sendo o G2 o grupo que mais evidenciou isto, conforme mostrado na Tabela 2.

Portanto, merece destaque o achado de que estes sujeitos pertenciam ao grupo que recebeu o tratamento com laser placebo. A percepção de 100% de melhora funcional para falar alguma palavra e sentir o sabor dos alimentos pode ter tido influência deste efeito. Resultados positivos no quadro funcional dos indivíduos, como na mobilidade mandibular, também já foram vistos^(18)^, bem como em outra pesquisa que identificou maior redução de dificuldade para mastigar nos sujeitos tratados com exercícios miofuncionais orofaciais associados ao laser placebo^(17)^.

Estas evidências existem porque a resposta ao placebo é um evento neurobiológico – cuja atividade em áreas corticais tem sido associada com a inibição da dor e centro afetivo e cognitivo^(28)^. A expectativa do paciente, um bom relacionamento entre paciente-terapeuta, associados com a sensação de estar recebendo um tratamento mais completo pela presença tecnológica do laser podem justificar essa possibilidade^(29)^.

Diferente dos achados neste estudo, uma pesquisa realizada com 11 mulheres com DTM muscular, dividida em dois grupos, um experimental (GE) submetidas à TMO associada à fotobiomodulação, e outro Controle Positivo (GC), submetidas à TMO associada à fotobimodulação inativa (placebo), com a intervenção sendo realizada também em 10 sessões, com comprimento de onda 830 nm, fornecendo a dose pontual de 3J. O GE teve aumento nas medidas dos movimentos de abertura e de protrusão mandibular, e evidenciou melhora na avaliação da QV^(30)^. Demonstrando resultados mais expressivos quando as sessões mantiveram a dose (3J), diferente do critério utilizado nesta pesquisa que variou as doses com 6J da 1ª a 5ª sessão, com a finalidade de analgesia do quadro doloroso, e a partir da 6ª sessão com a dose de 4J, para bioestimular os ganhos funcionais promovidos pela terapia fonoaudiológica.

Isto trouxe a reflexão de que apesar do laser de baixa potência propor diversos mecanismos de ação no organismo e ser referido positivamente nas intervenções com tratamentos associados de pacientes com DTM^(27)^, sua eficácia clínica apresenta-se em diferentes parâmetros, doses e critérios metodológicos nos estudos. Dessa forma, os efeitos da fotobiomodulação na otimização da terapia fonoaudiológica ainda precisam ser mais evidenciados.

Diante do exposto, a realização dessa pesquisa permitiu fortalecer o trabalho desempenhado pelo fonoaudiólogo no acompanhamento de pacientes com DTM através da Terapia Miofuncional Orofacial, uma vez que ela é de competência desse profissional e considerada a referência na área de Motricidade Orofacial. Porém, algumas limitações neste estudo foram identificadas, como número amostral pequeno e a diferença do número de participantes entre os grupos – o que pode ter favorecido para que a hipótese da pesquisa não tenha sido respondida, que pessoas com disfunção temporomandibular muscular submetidas a fotobiomodulação associada a TMO teriam mais ganhos na qualidade de vida do que as mulheres com as mesmas características submetidas só a TMO.

Sendo assim, sugere-se a continuidade das discussões científicas com o uso desse recurso no campo da fonoaudiologia, explorando as análises com amostras maiores para que se obtenha evidências científicas e consolidação do conhecimento acerca dos efeitos da fotobiomodulação na qualidade de vida em pessoas com DTM.

## CONCLUSÃO

As pessoas com disfunção temporomandibular que receberam o tratamento de fotobiomodulação associada a terapia miofuncional orofacial perceberam melhora da qualidade de vida relacionada à saúde oral, assim como as tratadas com o laser placebo. Houve correlação positiva e forte em ambos os grupos na melhora do grau da dor e autopercepção da QVRSO.
